# Antenatal depressive symptoms and utilisation of delivery and postnatal care: a prospective study in rural Ethiopia

**DOI:** 10.1186/s12884-017-1383-8

**Published:** 2017-06-29

**Authors:** Tesera Bitew, Charlotte Hanlon, Eskinder Kebede, Simone Honikman, Michael N. Onah, Abebaw Fekadu

**Affiliations:** 1 Addis Ababa University, College of Health Sciences, School of Medicine, Department of Psychiatry, Addis Ababa, Ethiopia; 2 Debre Markos University, Institute of Educational and Behavioural Sciences, Department of Psychology, Debre Markos, Ethiopia; 3 King’s College London, Institute of Psychiatry, Psychology and Neuroscience, Centre for Global Mental Health, London, UK; 4 Addis Ababa University, College of Health Sciences, Department of Obstetrics and Gynecology, Addis Ababa, Ethiopia; 5 University of Cape Town, Department of Psychiatry and Mental Health, Alan J Flisher Centre for Public Mental Health, Perinatal Mental Health Project, Cape Town, South Africa; 6 King’s College London, Institute of Psychiatry, Psychology and Neuroscience, Department of Psychological Medicine, Centre for Affective Disorders, London, UK; 7 Addis Ababa University, Centre for Innovative Drug Development and Therapeutic Trials for Africa (CDT-Africa), Addis Ababa, Ethiopia

**Keywords:** Antenatal depressive symptoms, Delivery care use, Postnatal care use, Assisted delivery, Planned institutional delivery, Ethiopia

## Abstract

**Background:**

Uptake of delivery and postnatal care remains low in Low and Middle-Income Countries (LMICs), where 99% of global maternal deaths take place. However, the potential impact of antenatal depression on use of institutional delivery and postnatal care has seldom been examined. This study aimed to examine whether antenatal depressive symptoms are associated with use of maternal health care services.

**Methods:**

A population-based prospective study was conducted in Sodo District, Southern Ethiopia. Depressive symptoms were assessed during pregnancy with a locally validated, Amharic version of the Patient Health Questionnaire (PHQ-9). A cut off score of five or more indicated possible depression. A total of 1251 women were interviewed at a median of 8 weeks (4–12 weeks) after delivery. Postnatal outcome variables were: institutional delivery care utilization, type of delivery, i.e. spontaneous or assisted, and postnatal care utilization. Multivariate logistic regression was used to examine the association between antenatal depressive symptoms and the outcome variables.

**Results:**

High levels of antenatal depressive symptoms (PHQ score 5 or higher) were found in 28.7% of participating women. Nearly two-thirds, 783 women (62.6%), delivered in healthcare institutions. After adjusting for potential confounders, women with antenatal depressive symptoms had increased odds of reporting institutional birth [adjusted Odds Ratio (aOR) =1.42, 95% Confidence Interval (CI): 1.06, 1.92] and increased odds of reporting having had an assisted delivery (aOR = 1.72, 95% CI: 1.10, 2.69) as compared to women without these symptoms. However, the increased odds of institutional delivery among women with antenatal depressive symptoms was associated with unplanned delivery care use mainly due to emergency reasons (aOR = 1.62, 95% CI: 1.09, 2.42) rather than planning to deliver in healthcare institutions.

**Conclusion:**

Improved detection and treatment of antenatal depression has the potential to increase planned institutional delivery and reduce perinatal complications, thus contributing to a reduction in maternal morbidity and mortality.

## Background

Depressive disorders constitute a public health challenge that contributes a substantial proportion of years lived with disability, globally [1, 2]. In World Health Organization (WHO) Global health estimates, depressive disorders were single largest contributors to non-fatal health loss with more than 80% of this non-fatal disease burden in Low and Middle Income Countries (LMICs) [3]. In the general population, depression is associated with a number of adverse impacts, including poorer quality of life [4], physical ill-health [2, 4], reduced adherence to medical recommendations [5, 6], increased use of health care services [7] arising because of increased medically unexplained symptoms [5], reduced social support, increased worries [8], co-morbid illness [5], and increased health risk behaviours and reduced self-care [9].

Among perinatal women, depression is additionally associated with particular adverse public health consequences [2, 10–14] such as adverse effects on perinatal outcomes and the growth, health and development of the child [1, 15, 16], as well as adverse effects on maternal health. During pregnancy, depression has multiple negative effects independent of postnatal depression [1, 15, 16] and is highly prevalent, with estimates ranging from 10 to 20% [17–22] worldwide, while higher prevalence levels are reported in LMICs (20%–39%) [17, 18, 23–27]. Most maternal deaths (99% of global maternal deaths) occur in LMICs [28, 29] due to undetected and/or inadequately treated complications such as infection, haemorrhage, unsafe abortion, hypertension and obstructed labour [28, 30–33]. These complications are mostly preventable by improving the uptake of maternal health care services [34, 35] in general, and through the improvement of skilled delivery in health care facilities, in particular [36–40]. The WHO has proposed skilled institutional delivery as a key strategy towards reducing maternal deaths across the globe [41].

Established factors associated with institutional delivery in Ethiopia are higher levels of education for the woman [34, 37, 38, 42–44] or her partner [29, 34], better socioeconomic status [43], urban residence [34, 37, 38, 43, 45, 46], previous experience of attending antenatal care [34, 37, 43, 47], increased women’s autonomy [48, 49], media exposure, and prior experience of obstetric complications [50, 51]. However, the impact of maternal depression on women’s use of maternal health care has received little attention in the literature. The authors were only able to identify one relevant study from Ghana [22] where no association was found between antenatal depression and uptake of institutional delivery, although the analysis did not control for important confounders, such as comorbid medical conditions and obstetric complications.

Investigating the potential impact of antenatal depressive symptoms on uptake of delivery care and postnatal care utilisation is essential to design future intervention strategies in LMIC settings, where perinatal outcomes are poor [52] and prevalence of antenatal depressive symptoms high [19, 21]. We hypothesized that antenatal depressive symptoms would, independent of socio-economic and demographic factors, reduce uptake of institutional delivery and postnatal care due to several behavioural features associated with depression: loss of motivation and interest in common activities including self-care [9]; reduced social support [8], increased cognitive deficit to make decisions and evaluate alternatives [53] and reduced adherence to healthcare practitioner recommendations [5, 6]. Thus, improved detection and treatment of antenatal depressive symptoms, albeit not prioritized [54], is hypothesized to shift these behavioural manifestations and thus increase rates of planned institutional delivery and improve maternal and perinatal outcomes.

Thus, this study aimed to investigate prospectively the association between women’s antenatal depressive symptoms, uptake of institutional delivery (planned and unplanned), and postnatal care utilisation after controlling for socioeconomic, demographic, obstetric and medical factors.

## Methods

### Study design and setting

A population-based, prospective study was conducted in Sodo District, located in the Southern Nations, Nationalities and People’s Region (SNNPR) of Ethiopia. The district has 54 rural and four urban sub-districts *(*“*kebeles”)*, the smallest administrative unit in Ethiopia. The official language of the region and the district is Amharic. An estimate of about 161,000 people (79,000 men; 82,000 women) resided in Sodo in 2007. The majority of the inhabitants belong to the Sodo Gurage ethnic group (85%), with the remaining population being mostly Oromo and Amhara in ethnicity [52]. Agriculture is the main economic activity within the region.

### Cohort identification

The cohort was formed by recruiting all consenting pregnant women in the district in their second and third trimester of pregnancy, between early September and end of November 2014. In the current Ethiopian healthcare system, community based health workers (Health Extension Workers, HEWs) are tasked with community health prevention and promotion activities. They are also tasked with identification and monitoring of pregnant women and to keep accurate and up-to-date maternal records in health posts (frontline primary healthcare facility staffed by HEWs).

In support of these activities, HEWs coordinate with the health development army, a community-based network of health education volunteers, each of whom covers five families. The members of the health development armies are required to notify HEWs of all pregnant women in their respective areas. In this study, HEWs, members of health development armies, kebele chairmen and pregnant women themselves acted as key informants to identify all pregnant women in their respective sub-districts. Through home to home visits of identified women, the data collectors obtained informed consent from potential participants and conducted the baseline interviews. A minimum of three visits were carried out before considering participants ‘unavailable’ or ‘unidentifiable’.

Eligibility criteria for participation in the study included: (1) being in the second or third trimester of pregnancy; (2) continuously resident in the area for a minimum of 6 months; (3) no hearing or cognitive impairment that would affect their capacity to communicate adequately; (4) giving informed consent.

### Sample size

Sample size was estimated using EpiInfo version 7 [55] assuming a statistical power of 80% with a two tailed 5% margin of error; 11.7% of institutional delivery utilization among women without antenatal depression [52]; a 10% difference between women with and without antenatal depressive symptoms, which was assumed to be clinically significant. A three to one ratio for women with and without antenatal depressive symptoms was used based on the rates of antenatal depression cited in the literature for LMICs settings [17–19, 23–25]. On this basis, the target sample size was 1174 pregnant women (294 with antenatal depressive symptoms and 880 women without antenatal depressive symptoms). However, all 1311 antenatal baseline participants (356 with antenatal depressive symptoms and 955 without antenatal depressive symptoms) formed the cohort for this study [56].

### Data collection and quality control

Data collection was conducted by trained interviewers using an Amharic version of the questionnaires. Forty experienced data collectors and four supervisors were trained for 2 days by the main coordinator of the study (TB) on administration of the instruments, objectives of the study and ethical issues. Training methods included lectures, demonstrations and role-plays. TB closely monitored and supervised the conduct of the study through weekly meetings with the data collectors and supervisors. Completed questionnaires were checked carefully for consistency, adherence to instructions and missing data, first by the supervisors and then by the coordinator and data entry clerks. The data were double entered using EpiData version 3.1 [55] while data collection was proceeding. Completed questionnaires deemed missing or inconsistent were returned back to data collectors for investigation and correction. Data reporting was made in adherence to a STROBE statement checklist [57] to maintain standard of reporting for cohort data.

### Measurement

#### Outcome variables

Main outcomes were whether delivery occurred in a healthcare institution or at home without skilled birth attendatnt; (2) whether delivery in a healthcare setting was planned or unplanned and (3) whether postnatal care was utilized. The outcome variables were prospectively assessed at a median of 8 weeks postpartum (interquartile range of 6–11 weeks postpartum) using the lay interviewer-administered questionnaires. The delivery setting was dichotomized into home delivery vs. institutional delivery. The method of delivery was dichotomised as spontaneous vaginal delivery (SVD) or assisted delivery (instrumental vaginal delivery or Caesarean Section). Women who delivered in health care institutions were also asked whether this had been planned, or had arisen “due to prolonged labour”, and or “due to referral linked to complications” (the latter two responses were subsequently coded as ‘unplanned’). Postnatal care utilization was rated positive if there was at least one visit to a health care professional within 4 weeks of delivery.

#### Primary exposure

The primary exposure was the occurrence of depressive symptoms antenatally. A locally validated Amharic version of the Patient Health Questionnaire (PHQ-9) [58] was used to screen for antenatal depressive symptoms at baseline, during the second and third trimesters of pregnancy. In studies from high-income countries, the cut-off scores indicating possible major depressive disorder cluster around 10 points [59], however, validation studies of the PHQ-9 in rural areas of low-income countries of sub-Saharan Africa, have found a lower optimal cut-off point. In Ethiopia, the PHQ-9 has been validated in antenatal women and in primary care settings in the neighbouring district of the current study, with the optimal cut-off point indicating probable depression identified as 5 or more in primary care attendees [60]. In a community sample of postnatal women in Ghana, the optimal cut-off to indicate probable depression was also 5 or more [22]. Therefore, in this study, a PHQ score of 5 or more was taken to indicate probable antenatal depression.

#### Potential confounders

Potential confounding variables were reviewed from the literature and assessed at baseline, during the second and third trimesters of pregnancy. A five item scale, the Women’s Abuse Screening Test (WAST) [61, 62] was used to assess intimate partner violence (IPV). WAST was chosen for its brevity and the acceptability of the wording. A score of one or more on WAST indicates women who have experienced IPV [61, 62]. A three item scale, the Oslo Social Support scale (OSS-3) [63], was used to assess social support. The OSS-3 scale has been used in a community based study in the same setting [64].

Questions from the 2011 Ethiopian Demographic Health Survey (EDHS) were used to collect information on previous stillbirth, spontaneous abortion, neonatal and infant mortality, and comorbid medical conditions, including HIV, tuberculosis, renal or cardiac diseases, hypertension, anaemia or gastritis. Pregnancy intention was coded as ‘intended’ if the woman intended the pregnancy to happen; ‘mistimed’ if the woman would have preferred the pregnancy to have happened at a future date and ‘unwanted’ if the woman did not want to be pregnant at all. The number of antenatal care (ANC) visits was also asked and it was adjusted to gestational age by dividing the number of actual ANC visits by expected number of ANC contacts for a given gestation [one, two, three and four ANC contacts were expected for women at 16th, 28th, 32nd and 40th weeks of gestation respectively based on WHO recommendations [41]]. Birth preparedness was also assessed using items taken from EDHS and other similar studies that asked mothers whether they had planned the means of transport, prepared a delivery kit, identified a health facility and obtained the money required for expenses during delivery [50, 52, 65].

A seven-item scale was used to assess the accessibility of health care facilities for women. This tool assessed the level of difficulty, distance to reach the nearest health facility, and travel time taken as well as affordability and availability of health facilities [66]. Pregnancy complications were assessed using a list of key danger signs during pregnancy as obtained from the EDHS [52]. Close-ended questions were used to assess socio-demographic and socio-economic variables, including residence, marital status, estimated monthly income and educational level of participants.

#### Data analysis

**Fig. 1:**
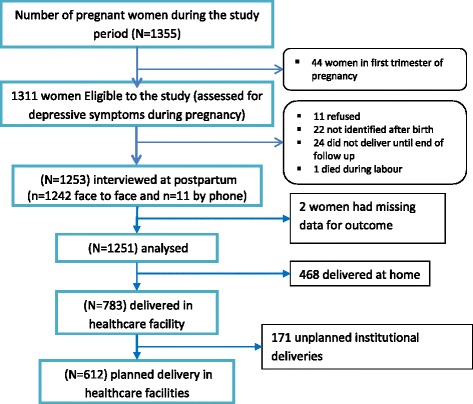
A diagram of sample recruitment procedure

Stata version 13.1 (Stat Corp, 2013) was used to analyse the data. Monthly income was divided into tertiles and labelled as “high”, “medium”, and “low” income categories. The profile of exposures and outcomes was described using simple descriptive summary values. The number of women lost to follow up was 4.6% including missing data in outcome variables of seven women (Fig. 1). Thus, complete case analysis was used as it was suggested that less than 5% lost to follow up was of little concern [67, 68].

Binary logistic regression was used to examine the association between antenatal depressive symptoms and the outcome variables. Bayesian Information Criterion (BIC) test was used to test model goodness of fit as a result of which ‘number of ANC visits’ was removed from two models. Socio-demographic and socioeconomic variables, interpersonal and life adversities (IPV, lack of social support), obstetric and medical conditions (experience of adverse perinatal outcomes, pregnancy complications, comorbid medical conditions, pregnancy intention, experience of institutional delivery and birth preparedness) were included in all analyses as potential confounders.

#### Ethical considerations

Ethical approval (ref. number: 024/14/psy dated 23/03/14) was obtained from the Institutional Review Board of the College of Health Sciences, Addis Ababa University. Women with a very high level of depressive symptoms (PHQ-9 ≥ 15) and those with suicidal ideation, as assessed by a particular item in the PHQ-9, were referred to health centres to access free primary care-based mental health services. Women with a PHQ-9 score above the validated cut off were not referred to health care facilities to avoid causing unnecessary concern among women with false positive symptoms, as the PHQ-9, as a screening tool, has low positive predictive validity [58, 69, 70].

## Results

From a baseline of 1311 pregnant participants, a total of 1251 women were prospectively interviewed (*n* = 1240 face to face and *n* = 11 by phone) about their delivery setting, method of delivery and postnatal care utilization with a response rate of 95.5% (Fig. 1). Women who were in the baseline sample were not significantly different from women who were in the follow-up sample with respect to selected baseline variables (Table 1).

**Table 1:** Characteristics of participants (*N* = 1251)

Characteristics		Baseline (*N* = 1311) N (%)	Followed Up (*N* = 1251) N (%)	chi2 (*p*-value)
PHQ Status	PHQ < 5	924 (70.5)	892 (71.3)	0.2098 (*p* = 0.647
	PHQ ≥ 5	387 (29.5)	359 (28.7)	
Marital Status	Married	1293 (98.6)	1235 (98.7)	
	single, divorced or widowed	18 (1.4)	16 (1.3)	0.0432 (*p* = 0.835)
Residence	Urban	103 (7.9)	101 (8.1)	0.0411 (*p* = 0.839)
	Rural	1208 (92.1)	1150 (91.9)	
Household Income^*^	High	459 (35.0)	438 (35.0)	
	Medium	423 (32.3)	397 (31.7)	0.1109 (*p* = 0.946)
	Low	429 (32.7)	416 (33.3)	
Mother’s Education	Non-literate	878 (67.0)	844 67.5)	
	Primary Schooling (Grade 1–8)	380 (29.0)	357 (28.5)	0.0714 (*p* = 0.965)
	Grade 9 & more	53 (4.0)	50 (4.0)	
Experience of Intimate Partner Violence	None	573 (43.7)	552 (44.1)	0.0453 (*p* = 0.831)
	One or more in life time	738 (56.3)	699(55.9)	
Pregnancy Intention	Wanted	734 (56.0)	701 (56.0)	
	Mistimed (wanted but not now)	102 (7.8)	93 (7.4)	0.1168 (*p* = 0.943)
	Unwanted	475 (36.2)	457 (37.3)	
Self-reported Pregnancy Complications	None	655 (50.1)	632 (50.5)	0.0594 (*p* = 0.808)
	One or more	656 (49.9)	619 (49.5)	
Chronic Illness	None	871 (66.4)	824 (66.4)	0.0001(0 = 0.994)
	One or more	440 (33.6)	416 (34.6)	
Previous institutional delivery	Yes	160 (12.2)	152 (12.2)	0.0018 (*p* = 0.967)
	No/primiparous	1151 (87.8)	1099 (87.8)	
Delivery Care Utilisation	Home delivery	--	468 (37.4)	--
	Institutional delivery	--	783 (62.6)	--
Type of Delivery	Normal vaginal	--	1115 (89.1)	--
	Assisted or operative	--	136 (10.7)	--
One or more Postnatal care Visits:	No	--	568 (45.8)	--
	Yes	--	672 (54.2)	--
Reason for use of inst. delivery	Planned	--	612 (78.3)	--
	Unplanned	--	171 (21.7)	--

Single Marital Status = unmarried, widowed, divorced

^*^Income was categorized into tertiles as low, medium and high

*CS* Caesarian Section

### Characteristics of the participants

Most women were married (98.7%), were rural residents (91.9%) and were illiterate (67.5%). Nearly two thirds of women (62.6%) delivered in health institutions. Over half of the women (55.6%) reported experiencing some form of intimate partner violence in their life time and 43.8% had an unintended pregnancy (7.3% mistimed and 37.3% unwanted) (Table 1).

### Antenatal depressive symptoms and institutional delivery

High levels of antenatal depressive symptoms (PHQ score 5 or higher) were found in 28.7% of participating women. In the adjusted model (Table 2), women with high levels of antenatal depressive symptoms had increased odds of delivering in health care institutions [adjusted Odds Ratio (aOR) =1.42, 95% Confidence Interval (CI): 1.06, 1.92] independent of pregnancy complications and comorbid medical conditions. Increased access to a health care facility (aOR = 1.10, 95% CI: 1.04, 1.16), primary level education (compared to no education) (aOR = 1.51, 95% CI: 1.10, 2.08), having a previous delivery in a health care institution (aOR = 2.21, 95% CI: 1.36, 3.58) and increased birth preparedness (aOR = 1.32, 95% CI: 1.20, 1.46) were also associated with increased odds of institutional delivery. Rural residence (aOR = 0.39, 95% CI: 0.18, 0.89) and increasing parity (aOR = 0.90, 95% CI: 0.84, 0.97) were associated with reduced odds of institutional delivery.

**Table 2:** Use of Institutional Delivery care as a function of antenatal depressive symptoms

Variables	Odds of Institutional delivery (vs. home) (*n* = 1251)		Odds of unplanned Institutional delivery (vs. Planned)(*n* = 783)	
	(cOR, 95% CI)	(aOR, 95% CI)	(cOR, 95% CI)	(aOR, 95% CI)
Depressive Symptoms: PHQ9 ≥ 5	1.09 (0.85, 1.41)	1.42 (1.06, 1.92)*	1.57 (1.10, 2.35)*	1.62 (1.09, 2.42)*
Access to Health Facility	1.16 (1.10, 1.22)**	1.10 (1.04, 1.16)*	0.97 (0.90, 1.04)	0.99 (0.91, 1.07)
Marital Status: Single	0.77 (0.28, 2.07)	0.70 (0.24, 2.06)	0.45 (0.06, 3.60)	0.37 (0.04, 3.10)
Residence: Rural	0.13 (0.06, 0.27)**	0.39 (0.18, 0.89)*	1.02 (0.60, 1.72)	0.78 (0.39 1.56)
Household Income:				
Low	1	1	1	1
Medium	1.13 (0.85, 1.49)	0.89 (0.65, 1.20)	1.44 (0.95, 2.19)	1.67 (1.07, 2.60)*
High	1.31 (0.99, 1.73)	1.05 (0.77, 1.45)	1.03 (0.68, 1.58)	1.37 (0.86, 2.18)
Educ. Level:				
Illiterate	1	1	1	1
Primary Schooling	2.25 (1.71, 2.95)**	1.51 (1.10, 2.08)*	0.70 (0.48, 1.02)	0.65 (0.41, 1.01)
≥ Secondary	19.01 (4.59, 78.71**	3.99 (0.87, 18.27)	0.73 (0.34, 1.55)	0.57 (0.22, 1.51)
Intimate Partner violence	0.97 (0.94, 1.01)	0.97 (0.93, 1.02)	0.98 (0.92, 1.04)	0.95 (0.89, 1.02)
Social Support	1.01 (0.95, 1.07)	0.95 (0.88, 1.01)	0.90 (0.83, 0.98)*	0.93 (0.85, 1.02)
Institutional Delivery of previous baby: Yes	3.62 (2.30, 5.68)**	2.21 (1.36, 3.58)*	1.15 (0.93, 1.43)	1.39 (0.85, 2.26)
Birth Preparedness	1.5(1.32, 1.56)**	1.32 (1.20, 1.46)**	0.81 (0.72, 0.92)*	0.81 (0.71, 0.93)*
Parity (Birth Order)	0.83 (0.79, 0.88)**	0.90 (0.84, 0.97)*	1.00 (0.92, 1.08)	0.93 (0.83, 1.03)
Pregnancy Intention:				
Wanted	1	1	1	1
Mistimed	0.62 (0.40, 0.96)*	0.70 (0.44, 1.11)	0.86 (0.42, 1.77)	0.87 (0.41, 1.83)
Unwanted	0.64 (0.51, 0.82)**	0.84 (0.64, 1.11)	1.03 (0.71, 1.48)	0.95 (0.63 1.43)
Symptoms of Pregnancy Comp.: ≥1	0.88 (0.70, 1.11)	0.88 (0.68, 1.15)	1.16 (0.83, 1.64)	1.01 (0.69, 1.47)
Comorbid medical conditions: ≥1	1.32 (1.04, 1.67)*	1.23 (0.95, 1.59)	0.99 (0.70, 1.39)	0.96 (0.67, 1.38)

*significant at <0.05, **significant at <0.001

Access to health care facility score: minimum = 5; Maximum = 15; Mean = 11.98; SD = 2.40

Birth Preparedness score: Minimum = 0; Maximum = 4; Mean = 1.61; SD = 1.42

Intimate Partner violence score: minimum = 9; Maximum = 16; Mean = 2.14; SD = 2.88

Social support score: minimum = 3; Maximum = 14; Mean = 10.68; SD = 2.00

Parity score: minimum = 0; Maximum = 12; Mean = 2.74; SD = 2.09

Among women who delivered in healthcare institutions, there was greater odds of unplanned institutional delivery, mainly due to emergency reasons such as prolonged labour and bleeding, among those with antenatal depressive symptoms (aOR = 1.62, 95% CI: 1.09, 2.42) (Table 2). Among other predictors, being in the medium income category (aOR = 1.67, 95% CI: 1.07, 2.60) was associated with increased odds of unplanned institutional delivery while each increment in birth preparedness score (aOR = 0.81, 95% CI: 0.81, 0.71, 0.93) was associated with reduced odds of unplanned institutional delivery as compared with planned institutional delivery.

### Method of delivery and antenatal depressive symptoms

After adjusting for potential confounding variables (Table 3), women with antenatal depressive symptoms had increased odds of assisted delivery compared to women without antenatal depressive symptoms (aOR = 1.72, 95% CI: 1.10, 2.69). Factors that remained significantly associated with increased odds of assisted delivery in the adjusted model were increased access to health care facilities (aOR = 1.11, 95% CI: 1.02, 1.22); having a secondary level education (aOR = 2.40, 95% CI: 1.02, 5.67) and having one or more symptoms of pregnancy complications (aOR = 1.54, 95% CI: 1.01, 2.34). Rural residence (aOR = 0.39, 95% CI: 0.21, 0.75) and increasing parity (aOR = 0.72, 95% CI: 0.62, 0.82) were associated with reduced odds of assisted delivery.

**Table 3:** Having assisted delivery and use of postnatal care vs antenatal depressive symptoms

Variables	Assisted delivery vs SVD		Postnatal care use	
	(cOR, 95% CI)	(aOR, 95% CI)	(cOR, 95% CI)	(aOR, 95% CI)
Depressive Symptoms: PHQ9 ≥ 5	1.31 (0.90, 1.91)	1.72 (1.10, 2.69)*	1.12 (0.87, 1.43)	1.13 (0.85, 1.50)
Access to Health Facility	1.18 (1.09, 1.29)**	1.11 (1.02, 1.22)*	1.05 (1.00, 1.10)*	1.01 (0.96, 1.06)
Marital Status: Single	2.79 (0.89, 8.76	2.33 (0.66, 8.17)	0.74 (0.27, 2.04)	0.71 (0.24, 2.16)
Residence: Rural	0.18 (0.11, 0.29)**	0.39 (0.21, 0.75)*	0.60 (0.39, 0.93)*	1.42 (0.82, 2.46)
Income per family:				
Low	1	1	1	1
Medium	0.91 (0.58, 1.42)	0.72 (0.45, 1.17)	0.97 (0.74, 1.28)	0.91 (0.68, 1.21)
High	1.06 (0.69, 1.62)	0.80 (0.48, 1.34)	1.12 (0.86, 1.47)	0.98 (0.72, 1.33)
Educational Level:				
Illiterate	1	1	1	1
Primary Schooling	2.62 (1.78, 3.88)**	1.29 (0.81, 2.06)	1.19 (0.92, 1.53)	1.0 (0.79, 1.43)
≥ Secondary	10.85 (5.84, 20.16)**	2.40 (1.02, 5.67)*	4.03 (1.93, 8.41)**	3.23 (1.34, 7.77)*
Intimate Partner violence	0.97 (0.91, 1.03)	0.95 (0.88, 1.02)	1.09 (2.04, 1.13)**	1.11 (1.06, 1.16)**
Social Support	1.00 (0.92, 1.09)	1.03 (0.93, 1.14)	1.01 (0.95, 1.06)	0.99 (0.93, 1.05)
Institutional Delivery of last baby: Yes	2.07(1.31, 3.27)*	1.40 (0.82, 2.37)	-	**--**
Number of Antenatal Care visits visits adjusted to Gestational Age	**--**	--	1.72 (1.40, 2.12)**	1.42 (1.14, 1.76)*
Birth Preparedness	1.21 (1.07, 1.36)*	1.01 (0.87, 1.17)	1.32 (1.21, 1.43)**	1.30 (1.18, 1.42)**
Parity	0.65 (0.58, 0.73)**	0.72 (0.62, 0.82)**	0.96 (0.91, 1.02)	1.01 (0.94, 1.08)
Pregnancy Intention:				
Wanted	1	1	1	1
Mistimed	0.62 (0.29, 1.31)	0.87 (0.39, 1.95)	1.27 (0.81, 1.99)	1.26 (0.79, 2.01)
Unwanted	0.54 (0.36, 0.82)*	1.01 (0.63, 1.62)	0.80 (0.63, 1.02)	0.86 (0.66, 1.13)
Symptoms of Pregnancy Comp.: ≥1	1.48 (1.03, 2.12)*	1.54 (1.01, 2.34)*	0.90 (0.72, 1.12)	0.85 (0.66, 1.10)
Comorbid medical conditions: ≥1	1.38 (0.96, 1.97)	1.47 (0.99, 2.19)	0.94 (0.74, 1.18)	0.84 (0.66, 107)

*significant at <0.05, **significant at <0.001

Access to health care facility score: minimum = 5; Maximum = 15; Mean = 11.98; SD = 2.40

Birth Preparedness score: Minimum = 0; Maximum = 4; Mean = 1.61; SD = 1.42

Intimate Partner violence score: minimum = 9; Maximum = 16; Mean = 2.14; SD = 2.88

Social support score: minimum = 3; Maximum = 14; Mean = 10.68; SD = 2.00

Number of ANC visits: minimum = 0; Maximum = 8; Mean = 1.524; SD = 1.50

Parity score: minimum = 0; Maximum = 12; Mean = 2.74; SD = 2.09

### Postnatal care utilization and antenatal depressive symptoms

Women having a secondary level education (aOR = 3.23, 95% CI: 1.34, 7.77), increased intimate partner violence (aOR = 1.11, 95% CI: 1.06, 1.16), increased number of antenatal care (ANC) visits (aOR = 1.42, 95% CI: 1.14, 1.76) and an increased birth preparedness score (aOR = 1.30, 95% CI: 1.18, 1.42) had increased odds of having postnatal care visits in both the univariate and adjusted models (Table 3).

## Discussion

In this prospective, population-based study from rural Ethiopia, 28.7% of pregnant women were screened to have symptoms of depression. These women had significantly increased odds of unplanned utilisation of institutional delivery care, mostly due to emergency presentations rather than planning to deliver in a healthcare facility. Assisted delivery was also significantly greater in women with antenatal depressive symptoms.

Overall, the prevalence of antenatal depressive symptoms in our study is consistent with studies in Ghana (26.3%) and Cote d’Ivoire (28.3%) which used the same measure [18, 23]. Studies in South Africa [25], Vietnam [17] and Southern Brazil [24] using other screening tools (Self-Reporting Questionnaire and Edinburgh Postnatal Depression Scale) also reported rates of antenatal depressive symptoms of 30–39%.

Prior to the introduction of the health extension programme [71], and until 2011, institutional delivery rates in Ethiopia were very low [52]. Recent trends indicate improving rates, especially in southern Ethiopia, with nearly 50% of women in community-based samples, and nearly three-quarters of women in urbanized areas delivering in health facilities [72–74]. Our study finding that over 60% of women deliver in an institution is in-line with this positive trend.

We hypothesized that antenatal depressive symptoms are associated with a reduction in women’s uptake of institutional delivery through reduced self-care, social support and reduced women’s adherence to healthcare practitioners’ recommendations. In contradiction with our hypothesis, we found a significantly increase in uptake of institutional delivery among women with antenatal depressive symptoms independent of pregnancy complications and comorbid medical conditions. Increased emergencies related to labour complications might explain this contradiction.

The increased odds of assisted delivery and uptake of unplanned institutional delivery among women with antenatal depressive symptoms supports others’ findings that women with depressive symptoms have an increased risk of labour complications [11, 75–77]. We found the main reasons for institutional delivery of women with antenatal depressive symptoms to be emergencies related to labour complications endorsed as ‘prolonged labour’ and or ‘referral due to labour complications’. Our findings reflect those of a study in Ghana where antenatal depression was associated with assisted delivery and other perinatal complications, including prolonged labour, vaginal tears, loss of consciousness, heavy vaginal bleeding, surgery to repair or remove the womb and blood transfusion [22]. Similarly, a previous community based cohort study in Ethiopia [14] and studies in China [78] and Ghana [22] demonstrated that antenatal depression was associated with prolonged labour or non-progressive preterm contractions [76]. Studies in Peru [79] and in Finland [80] also demonstrated increased odds of preeclampsia among women with antenatal depression while studies in California [77] and Canada [76] demonstrated increased odds of infection among this group of women. These perinatal complications are thus, more likely to increase unplanned institutional delivery and assisted delivery among women with depressive symptoms. There are also biological explanations for the association of depression with perinatal complications. In these explanations, depression is purported to hyper stimulate Hypothalamus-Pituitary-Adrenal (HPA) axis to produce hormones that have adverse effects on the uterine environment [81–83].

Furthermore, studies have demonstrated that women with antenatal depressive symptoms are more likely to have somatic complaints, co-morbid medical conditions [6, 84] and other perinatal complications [12, 14, 85, 86] as well as increased fear of childbirth and worries about death and survival during labour [8, 87] which may increase the likelihood of institutional deliveries in this group.

Our study showed that rural residence was associated with reduced odds of institutional delivery and reduced odds of having assisted delivery, while increased access to health care was associated with increased odds of institutional delivery and having an assisted delivery. These results support consistently findings from others’ work [34, 37, 38, 43, 45, 46, 73, 88, 89]. Increased parity was associated with reduced odds of institutional delivery and assisted delivery, which is also consistent with other local studies [45, 46, 88, 89]. This may be due to decreased likelihood of prolonged labour with increase in parity.

The strength of our study is that it was prospective and used a locally validated measure of depressive symptoms. Selection bias was also minimized in this population-based study as a robust method was used to identify all cases during the study period. However, we cannot be entirely certain if all eligible participants in the district were identified during the time period of the study. Respondent recall bias was minimized by measuring outcome variables within 6–11 weeks of birth. Furthermore, a broader range of confounders such as pregnancy complication and comorbid illnesses were controlled in our models.

Our study did not however, consider the cultural beliefs of women regarding seeking postnatal care. This is pertinent in rural Ethiopia where women are not encouraged to leave their homes until 2–3 weeks postpartum. This might explain the non-significant finding in the association between antenatal depressive symptoms and postnatal care utilization. Because of the low predictive value of PHQ-9 [69, 70], as a screening tool, it is likely that a proportion of PHQ-9 positive women do not meet criteria for a diagnosis of depression. Nonetheless, we found an important association of antenatal depressive symptoms with maternal health care utilization.

## Conclusion

Among women with antenatal depressive symptoms, there was increased odds of institutional delivery in general, and increased unplanned institutional delivery utilisation mainly due to emergency reasons. Assisted delivery was also increased among this group of women, which might have been due to labour complications. Thus, improved detection and treatment of antenatal depression has the potential to increase planned institutional delivery and reduce perinatal complications, thus contributing to a reduction in maternal morbidity and mortality, as well as improved neonatal health.

## Abbreviations

ANC: Antenatal Care
aOR: adjusted Odds Ratio
CI: Confidence Interval
cOR: crude Odds Ratio
CS: Caesarian Section
LMIC: Low and Middle Income Countries
SVD: Spontaneous Vaginal Delivery
WHO: World Health Organization
